# Progress in Surgery

**Published:** 1899-02-11

**Authors:** 


### Progress in Surgery.

### ABDOMINAL SURGERY.

**Non-penetrating Wounds** of the abdomen may cause serious mischief, and earlier operative treatment is more frequently resorted to than formerly. In more than half of the fatal cases of abdominal contusion the spleen has been found to be injured. Rupture of the intestine is necessarily very fatal; thus of seven cases operated on only one was saved by operation, although this represents a clear gain to surgery.[1] Rupture of the kidney or bladder may usually be diagnosed with comparative ease, but some injuries baffle diagnosis, and a small exploratory incision may be demanded. Of four cases recorded by Marmaduke Shield, one was successful, the condition found being hæmorrhage from a mesenteric vein.[2]

**Penetrating Wounds** are as a rule subjected to exploratory operation. Vulliet has analysed three hundred and fifty-five cases, and comes to the following conclusions, amongst others: Exploratory laparotomy must be performed at once; intestinal perforation cannot be diagnosed by symptoms; peritonitis, when once established, nearly always ends fatally; shock does not contra-indicate operation, it may be due to hæmorrhage.[3] A truly remarkable case has been reported by Captain George Bidie, in which one and a half inches of gut protuded from an incised wound of the abdomen, the gut having been smeared over with cow dung by the natives, according to their wonted custom. The gut was cleansed, repaired, and returned, and the boy recovered.[4]

**Abdominal Incisions.**—It is now customary with the best surgeons to separate muscular fibres rather than to divide them, and to preserve the nerves supplying them. By these means, together with aseptic operation, ventral hernia is much less common than formerly.

**Gastrostomy.**—The Kader-Senn method of performing this operation is strongly advocated by A. E. Maylard with a record of two successful cases. It consists in drawing out a cone of stomach through the parietal incision, passing two parallel purse-string sutures round the base of the cone to constrict it, and then sewing the gastric wall to the parietes. In the second stage an opening is made into the apex of the protruded gastric wall and the gastric coats inverted. The inversion is aided by supporting the orifice on an india-rubber tube temporarily placed. The advantages of the operation are its simplicity, rapidity, and freedom from subsequent leakage. Whether the inverted valve functionally persists for long afterwards remains to be seen. The cases recorded speak of an absence of leakage after two months.[5] Fenton Twick employs a somewhat similar method operating in the following stages: (*a*) Folding the anterior wall of the stomach, and securing it by three stitches; (*b*) bringing this fold together around the cannula and stitching it there; (*c*) inserting a guide thread at a selected point and passing it through the cannula; (*d*) bringing up a fold around the end of the trocar and stitching it over the ring; (*e*) suturing the parts and pushing the trocar and cannula into the stomach, leaving the latter in situ.[6] From his own personal experience, Mr. Paul Swain prefers to do gastrostomy by the method of Albert, in which a cone of the gastric wall is drawn through a subcutaneous side flap after union with peritoneum.[7]

**Gastro-enterostomy.**—The tide of fashion is setting towards posterior gastro-enterostomy. Two great advantages seem to exist, one is that the jejunum is not so kinked, and the other is that if a Murphy button be used it will tend to fall back into the jejunum, and not into the stomach. Mr. A. E. Barker mentions two very successful operations thus performed by the Murphy button.[8] Mr. C. B. Lockwood prefers to use sutures, and speaks well of the method of direct union of all three coats of the bowel successively, first sewing the three layers on the side away from the surgeon, and then those on the near side.[9]

The mortality of gastro-enterostomy in the hands of Mikulicz has been about 30 per cent. Collapse is the commonest cause of death, and kinking and spur-formation come next.

**Pylorectomy for Cancer** is rarely advocated owing to its mortality, but Dr. J. A. Adams urges earlier exploration and pylorectomy if possible, supporting his arguments by two cases, one of which was successful and lived 20 months afterwards.[10]

**Liver.**—The frequency with which an abscess of the liver may be traversed by an exploring aspirator without revealing pus is noted by A. Powell,[11] who, therefore, urges incision as a preferable measure. In one case the abscess was so large as to simulate ascites, and in another abscess round worms were found in the cavity. A dysenteric liver abscess is recorded by Dr. Frank Arnold, successfully treated through the pleura, aided no doubt by the existence of pleural adhesions.[12]

Two cases of resection and suture of liver for hydatid cyst have recently been described by Jacomet and by Palleroni. In the one the cyst was pedunculated and attached to the Spigelian lobe; in the other the cyst was excised and the liver sutured.[13]

**Gall-stones.**—One of two successful cases recorded by Mr. Bilton Pollard[14] resulted in the extraction of 443 stones from the gall-bladder, and a stone in the duct was needled and crushed.

Removal of gall-stones by the duodenal route was devised six years ago by McBurney, of New York. The anterior wall of the duodenum was incised and the orifice of the duct dilated until the stone was removed and bile flowed freely away. He has performed the operation six times[15] and finds it much easier when the stone is situated at the inner third of the duct, and regards the operation as quicker, cleaner, and safer. At the annual meeting of the British Medical Association in Edinburgh, Mayo Robson described three cases of operation by this method.[16] As in all jaundiced cases, there is great liability to hæmorrhage. Robson usually administers calcium chloride for some days prior to operation.

**Pancreas.**—Cysts of the pancreas have received more attention of late, and their pathology is now more intelligible. Payr has described such a cyst following an injury, and originating in the lesser bag of peritoneum passed through the foramen of Winslow into the greater sac. The fluid was brownish, alkaline, and contained albumen, sugar, mucin, and amylolitic ferment.[17] In a case under the care of Tiffany the fluid was of chocolate colour, neutral, of specific gravity 1,028, sterile. Sugar and bile acids were absent, but albumin, globulin, and propeptone were present. It presented marked proteolytic, amylolytic, and adipolytic properties. These cysts appear to arise commonly from the tail end of the pancreas, and first come to occupy the lesser sac of peritoneum, pushing the stomach upwards and the transverse colon downwards. The rate of growth may be from a few weeks to several years. In the majority of cases of supposed pancreatic cyst the fluid does not show proteolytic and amylolytic properties, so that the feature is not to be relied upon. A clear fluid may suggest hydatid origin, a bloody fluid an ovarian cyst. Simple puncture is dangerous, both from risk of puncturing the stomach and from risk of extravasation of the fluid. Perhaps the most reliable diagnostic signs are the presence of the digestive ferments and the discharge from the operation wound of detritus, which shows on section serous glandular tissue in the periphery, like that of the pancreas.

The prognosis of pancreatic abscess is grave, nevertheless the best treatment undoubtedly is to open the abscess, first stitching the wall of the abscess very carefully to the parietes, and then opening it.

**Intestinal Surgery.**—Mr. Frederick Treves remarks, what surgeons of experience already know, that "the symptoms which mark the onset of a case of acute intestinal obstruction have little or no diagnostic character. They are symptoms which attend all sudden and intense painful impressions upon important visceral nerves, and have been collectively described under the title of peritonism." In later stages individuality appears, and finally the patient dies poisoned.[18] Treves speaks favourably of his experience of Murphy's button, and of suturing the gut first by a continuous suture for mucous membrane, and afterwards interrupted Lembert sutures for the other coats.

Mr. W. Bennett prefers intestinal anastomosis—short-circuiting—to colotomy in cases of malignant disease, and regards Senn's plates as the best mode of treatment.[19] Mr. Battle has described three cases of obstruction of the bowels from malignant disease in which he excised the involved gut. In one case he excised secondarily the apex of a growth and intussusception per rectum. In the second case he made an artificial anus, and subsequently excised the growth. In a third case a sigmoid growth was excised primarily.[20] It may be well to refer to some points in an address by the late Greig Smith.[21] He called attention, in carcinoma of the bowel, to the excessive peripheral fatty tissue, and to the presence of a curious growth outside the cicatrising elements. Starting from the growth, the gut is contracted in all directions. He described the method of procedure which he devised, the first step consisting of fixation of the bowel and tumour in the parietal incision, the second in resection of the growth, suturing the divided ends of the bowel and returning them inside the parietal incision, but not into the general peritoneal cavity. This is the ideal method.

A series of five cases of lateral anastomosis for intestinal obstruction has been given by Dr. S. H. Hume,[22] comprising a lateral anastomosis between the cæcum and sigmoid, between the cæcum and transverse colon, between the sigmoid and colon, and two between the ileum and colon.

In cases of obstruction and trouble from chronic strictures of the rectum, non-malignant as well as malignant, Kelsey advocates excision of the rectum rather than colotomy, and thinks the mortality in experienced hands of Kraske's operation should not exceed 5 per cent. He is, moreover, accustomed to treat the appendages in women when necessary through the same incision.

Numerous cases of obstruction by bands have lately been recorded, but, though presenting varieties, they have a common type and treatment.

**Tuberculous Disease** of the intestine appears to be commonest in the cæcum and adjoining ileum and ascending colon. A cæcal case has been recorded by Dr. Lediard.[23]

**Appendicitis.**—M. Dieulafoy argues that appendicitis is not only infectious, but actually toxic, often producing far-reaching symptoms out of all proportion to the original disease. He also has noticed many cases of appendicitis which have given symptoms suggestive of renal colic.[24]

**The Chances of Life** in severe abdominal states can never be determined; one should always act on even a remote possibility of saving life. Lockwood says that there is a prospect of saving 50 per cent. of bad cases of peritonitis. One of the most remarkable examples of the maxim, "while there is life there is hope," is that recorded by Dr. E. T. Davies, of Liverpool. He was called to a woman who had prolapse of the intestines for over twenty-four hours, lying on straw and amongst filthy rags. He washed the guts, replaced them, but such was the irony of fate—the woman recovered.[25]

[1] Indian Med. Rec., Oct. 16, 1898. [2] Practitioner, Nov., 1898. [3] Med. Rec., Sept. 17, 1898. [4] Brit. Med. Jour., Sept. 24, 1898. [5] Lancet, Aug. 20, 1898. [6] Brit. Med. Jour., Nov. 16, 1898. [7] Lancet, Aug. 20, 1898. [8] Lancet, Nov. 12, 1898. [9] Lancet, Sept. 17, 1898. [10] Glasgow Med. Jour., Oct. 1898. [11] Med. Chron., Aug., 1898. [12] Lancet, July 30, 1898. [13] Epit. Brit. Med. Jour., Sept. 24, 1898. [14] Practitioner, Nov., 1898, p. 513. [15] Annals of Surgery, Oct., 1898. [16] Brit. Med. Jour., Nov. 5, 1898. [17] Epit. Brit. Med. Jour., Sept. 17, 1898. [18] Lancet, Nov. 5, 1898. [19] Practitioner, Nov., 1898. [20] Lancet, Nov. 12, 1898. [21] Lancet, July 23, 1898. [22] Lancet, Sept. 10, 1898. [23] Lancet, Aug. 13, 1898. [24] Epit. Brit. Med. Jour., Nov. 12, 1898. [25] Lancet, July 9, 1898.

